# The Lungs

**Published:** 1876-11

**Authors:** 


### THE LUNGS

Of a common man contain about one hundred and seventy millions of little bladders, or air cells, or little holes of different sizes, as in a sponge, and if these were cut open, and spread out, they would cover a space thirty times greater than the man's skin would; over one side of this vast surface the blood is spread out, by means of very small blood vessels; on the other side the air is diffused, and the substance of these little bladders is so thin, that the blood and air, in effect, come in contact, and the result of this contact is purification, heat and life; and death is the result, if this contact is prevented for three minutes; the reader will feel, therefore, how great is the necessity for a constant and full supply of pure air to the lungs. Hence the reason that those who live out of doors the most, live the longest, other things being equal. Of the 120,000 who die every year in England and Wales of Consumption, the greater number is among in-door laborers. This is the reason too, why the families of the rich in cities, soon become extinct; in summer they stay in the house to keep out of the sun, and in the winter to keep out of the cold; their faces are pale, their skin is flabby, and their limbs are weak; a young girl is put out of breath if she runs across the street; and seldom a day passes without a complaint of a headache or bad cold, or chilliness, or want of appetite, while the old father and mother of sixty winters or more, who lived in log cabins, cutting wood, hoeing corn, building fences, mauling rails, feeding cattle, spinning flax, weaving jeans in the old loom house during the day, and knitting socks in the chimney corner at night, going to bed a little after dark, and getting up to work before day, *they* scarcely know what an ache or a pain is, can eat heartily three times a-day, and are sound asleep in five minutes after the head reaches the pillow, and what is perhaps better, are always forbearing, good natured, cheerful, hospitable and kind, while their city progeny are poor, helpless, fretful, complaining invalids; heirs to millions they may possibly live to inherit for a brief period, but never can enjoy.

A tall man will take in at a full breath nine pints of air, while in ordinary breathing he takes in one pint, or forty cubic inches. If he be all at once deprived of this whole 40 inches, he will die in three minutes, and if death results from a total deprivation, an injury to health and life must take place in proportion as the amount breathed is less than forty inches; for example, of a hundred letter-pressmen, working in a room having less than 500 cubic feet of air to breathe, thirteen per cent had spitting of blood induced; while as many men having more than 600 feet, gave only four per cent of spitting blood; showing that, that most fatal symptom of Consumption is brought on in proportion as men breathe less pure air than health requires; the effect being the same whether there are not lungs to receive it, or whether there be not the air to be received.

It is with food as with air; a person soon dies if wholly deprived of either, but will gradually and a long time linger, if not quite enough is given for the wants of the system; and all are familiar with the fact, that consumptives gradually die as the lungs, by decay, become less and less able to receive the due amount of air.

